# Erdheim-Chester Disease: A Rare Presentation of a Rare Disease

**DOI:** 10.1177/2324709616663233

**Published:** 2016-08-22

**Authors:** Melissa Matzumura, Javier Arias-Stella, James E. Novak

**Affiliations:** 1 Detroit Medical Center/Wayne State University, Detroit, MI, USA; 2 Henry Ford Hospital, Detroit, MI, USA

**Keywords:** Erdheim-Chester disease, osteosclerosis, hairy kidney, retroperitoneal xanthogranuloma

## Abstract

Erdheim-Chester disease (ECD) is a rare, xanthogranulomatous, non-Langerhans cell histiocytosis with frequent systemic involvement. Although the diagnosis is based on characteristic histological and radiological findings, its identification can be challenging because of its heterogeneous presentation. Osteosclerosis of long bones, often associated with bone pain, is the most common initial manifestation, followed by extraskeletal manifestations in approximately 50% of cases. There is no standard treatment for ECD, although recommendations have been made on the basis of small studies. A systematic approach to the diagnosis of ECD is important, because its manifestations may be life-threatening and may require specific management. We report an atypical presentation of ECD, with early cardiac, renal, and central nervous system involvement, and only late skeletal manifestations.

## Introduction

Erdheim-Chester disease (ECD) is a rare, systemic, non-Langerhans cells histiocytosis of unknown origin characterized by xanthogranulomatous infiltration of foamy histiocytes surrounded by fibrosis.^1-4^ The first case was reported in 1930 by Jakob Erdheim and William Chester. ECD can affect virtually any organ, although the most common initial manifestation is osteosclerosis of long bones, which is associated with bone pain in 50% of cases.^5^ Approximately half of the patients also present with extraskeletal manifestations, of which central nervous system (CNS) involvement is a poor prognostic indicator. The heterogeneous clinical manifestations of ECD are determined by the type and degree of organ involvement, which makes its diagnosis challenging. The 2 major diagnostic criteria are typical clinicopathologic and radiographic findings.^1-3^ The most relevant characteristics of ECD are described in Table 1.

**Table 1.** Characteristics of Erdheim-Chester Disease. Both Histologic and Clinical Criteria are Required for Diagnosis.

Definition	Multisystemic non-Langerhans histiocytosis of unknown origin
Population	Middle-aged patients, slight male predominance
Histology	Foamy or “lipid-laden” macrophages or histiocytes surrounded by fibrosis
Most common findings	Symptomatic or asymptomatic osteosclerosis of long bones (most common initial manifestation)
	Retroperitoneal involvement, associated with renal failure and/or renovascular hypertension
	Peri-aortic infiltration
	“Hairy kidney”
	Central nervous system involvement (diabetes insipidus, exophthalmos, seizures, ataxia, headache)
	Pulmonary involvement
	Pericardial involvement
Diagnostic criteria	Foamy histiocyte infiltration and fibrosis or xanthogranulomatosis, with positive CD68 and negative CD1a
	Characteristic skeletal abnormalities (symmetric cortical osteosclerosis of diaphysis and metaphysis of long bones by plain radiography and/or abnormal intensity labeling of distal ends of the long bones shown by ^99^Tc bone scintigraphy)

Awareness of ECD and a systemic approach to its diagnosis is important, because its manifestations may be life-threatening and treatment may improve prognosis.

## Case presentation

**Figure 1.**
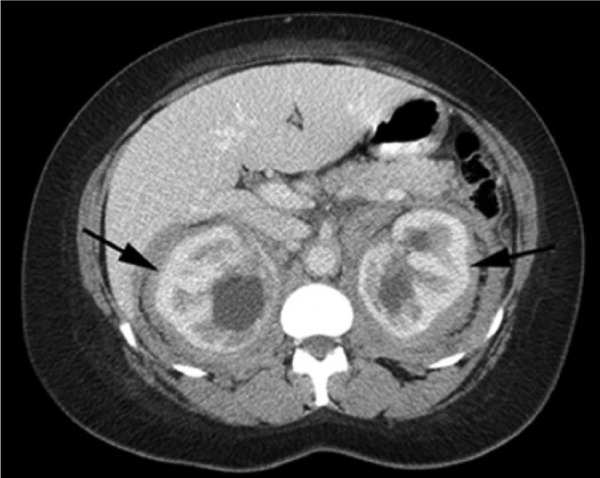
Radiologic findings. Computed tomography of the abdomen revealed soft tissue surrounding the aorta and kidneys, associated with inflammatory changes extending into the perirenal fascia bilaterally. Hydronephrosis was also present (arrows).

A 47-year-old African American woman with a history of poorly controlled hypertension was admitted for hypertensive emergency, acute kidney injury, right-sided abdominal pain, and progressive bilateral lower extremity edema. An abdominal computed tomography (CT) scan revealed soft tissue surrounding the aorta and both kidneys, associated with inflammatory changes extending into the perirenal fascia and moderate hydronephrosis (Figure 1). The left perinephric tissue was biopsied, and pathology demonstrated pericapsular adipose tissue with no evidence of malignancy or retroperitoneal fibrosis. Immunostains for melan A, HMB 45, KP1, smooth muscle actin (SMA), and pancytokeratin were negative, ruling out angiomyolipoma, renal cell carcinoma, or other epithelial malignancy. Autoimmune workup, including rheumatoid factor, antinuclear antibody, complement levels, anti–double-stranded DNA antibodies, and anti-topoisomerase, was similarly unremarkable. Serum IgG4 level, urine protein electrophoresis, and hepatitis panel were normal. Sedimentation rate, white blood cell count, and platelet count were persistently elevated all throughout the patient’s hospital course.

**Figure 2.**
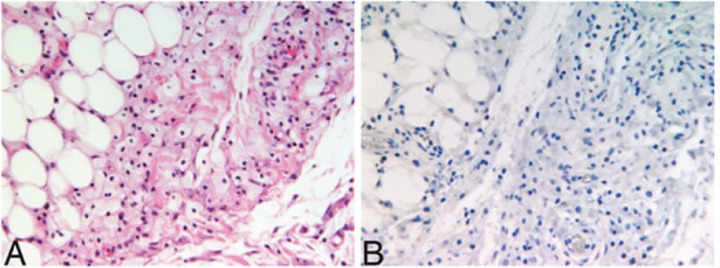
Photomicrographs of perinephric mass biopsy. (A) Adipocytes are observed toward the left of the field and histiocytes toward the center and right with no neutrophils or plasma cells and no nuclear atypia (hematoxylin and eosin, magnification 200×). (B) Negative CD1a immunostain (magnification 200×).

She was readmitted multiple times due to hypertensive emergency, acute renal failure, heart failure exacerbation, and hypercapnic respiratory failure. Over a period of 3 years, the patient underwent an extensive workup with no new significant findings. Specifically, she underwent 2 more perirenal biopsies, the latter of which showed perirenal fibrosis with foamy histiocytosis. Immunostains for S100, SMA, and pancytokeratin were again negative, as was Congo red staining for amyloid. The immunostain for CD68 was positive (Figure 2). A kidney Doppler ultrasound was negative for renal artery stenosis but suggested impaired perfusion bilaterally. Hematological evaluation included a normal peripheral blood smear and a bone marrow biopsy, which demonstrated normal cytogenetics, notably no Philadelphia chromosome, and no JAK2 V617F mutation. Bone scan showed age-appropriate bone mineral density, and femoral and humeral radiographs were negative for osteosclerosis.

The patient was suspected to have an atypical presentation of IgG4 disease with retroperitoneal fibrosis, and she was begun on high-dose prednisone, resulting in improved white cell count and creatinine. After 2 months of prednisone, follow-up abdominal CT showed a mild decrease in the size of the soft tissue mass in the posterior perirenal space. Subsequently, the patient was treated with mycophenolate mofetil as a steroid-sparing agent; however, both medications were discontinued after several months due to noncompliance and recurrent infections.

Dialysis was initiated due to worsening kidney function. Kidney biopsy revealed focal tubulointerstitial calcification and perinephric fibrohistiocytic proliferation. Immunostains, including CD34, S100, and beta-catenin, were negative, but SMA and CD68 were positive. Immunofluorescence for IgG, IgA, IgM, C1q, C3, C4, fibrinogen, albumin, and kappa and lambda light chains were all negative.

Three years after her initial presentation, the patient developed nonconvulsive seizures. Head CT demonstrated a hyperattenuating, extra-axial lesion overlying the left parietal lobe and intra-axial focal hypodensity, associated with diffuse, lobular, hyperattenuating soft tissue surrounding both optic nerves. Head magnetic resonance imaging (MRI) revealed a large focus of increased fluid attenuation inversion recovery (FLAIR) and T2 signal within the left temporoparietal lobe, multiple areas of increased FLAIR signal within the sulci of the frontal lobes, and multiple foci of increased signal scattered throughout the cerebral hemispheres. Also, there were areas of focal, dural, mass-like thickening overlying the left parietal and frontal lobes. Cerebral spinal fluid studies were negative for viral and bacterial infection and cytology was negative for malignancy, but total protein was slightly elevated. Brain biopsy was unable to be obtained.

Repeat bone radiography done 4 years after the initial normal X-ray showed sclerosis and cortical thickening involving bilateral radial and ulnar diaphyses and bilateral tibias and fibulas. BRAF oncogene testing was negative.

Based on clinical, radiographic, and immunohistological findings, the patient was diagnosed with ECD. Treatment with interferon (IFN) was not recommended due to recurrent severe infections. Unfortunately, due to multiple medical complications, including sepsis, respiratory failure, and end-stage renal disease, the patient expired.

## Discussion

ECD is a rare, systemic, and aggressive non-Langerhans form of histiocytosis, characterized by xanthomatous or xanthogranulomatous infiltration of foamy histiocytes and associated fibrosis but with no evidence of malignancy.^1,4,6,7^ Until 2014, there were approximately 500 reported cases of ECD. The etiology and pathogenesis continue to be poorly understood, but due to the presence of a V600E BRAF mutation in 54% of cases, a clonal neoplastic origin is suspected. Inflammation also plays a role in pathogenesis, with increased levels of IFN-α and interleukins.^1,2,6^ The differential diagnosis of non-Langerhans cell histiocytosis includes juvenile or adult-onset xanthogranuloma, xanthoma disseminatum, and Rosai-Dorfman disease.^8^

ECD most frequently affects men between the fourth and seventh decades, but pediatric cases have also been reported.^1,6,8^ Its diagnosis is challenging owing to its heterogeneous presentation, which depends on the type and degree of organ involvement. ECD may be asymptomatic or may present as a severe, multisystemic disease with life-threatening manifestations.^2^ The diagnostic criteria for ECD are included in Table 1.^1,3,4,6,8^ In a case series of 53 patients with ECD, 96% had skeletal involvement but only 50% presented with bone pain.^1^ Almost universally, skeletal involvement is the most common initial presentation, but in our case it was a late finding, occurring after cardiac, renal, and neurological involvement had progressed to their final stages.

Approximately half of the cases of ECD present with extraskeletal manifestations, including foamy histiocyte infiltration and fibrosis of the retroperitoneum, kidney, pericardium, orbit, CNS, lung, and skin.^1,2,4,8,9^ Two characteristic features of ECD, which were noted in our patient, are “hairy kidney” (symmetrical and bilateral dense infiltration of the perinephric space) and “coated aorta” (circumferential sheathing of the aorta).^1,6^ Renal failure may be secondary to either external compression by fibrotic tissue or infiltration of the parenchyma. Renal artery encasement may manifest as renovascular hypertension, which can be refractory to treatment, as in the current case. The most common cardiovascular manifestation is coated aorta, but patients may also develop pericardial effusions, heart failure, and valvular disease. In a case series of 72 patients, 56% had periaortic fibrosis, 44% had pericardial fibrosis, 30% had myocardial involvement, and 9% had valvular disease.^4^

Retroperitoneal involvement is also a common feature of ECD, affecting 30% to 50% of patients.^1,7^ Histiocytic infiltration and fibrosis can produce ureteral obstruction and hydronephrosis, which, as in the present case, can result in kidney injury requiring ureteral stenting.^6^ The presence of foamy cells, hairy kidney, and coated aorta, as well as the absence of plasma cells, vasculitis, and pelvic ureter and inferior vena cava involvement help differentiate ECD from primary retroperitoneal fibrosis.^1,4,6^

CNS involvement occurs in 25% to 50% of cases.^10^ CNS disease is an independent predictor of death^1,3,6,9,11^ and can be present even without the classic skeletal or visceral involvement.^8^ Three presentations are characteristic: infiltrative parenchymal, meningeal, and neurodegenerative lesions.^8,11^ Parenchymal disease is the most common and usually presents as neuroendocrine dysfunction, with diabetes insipidus the most common and sometimes the initial presentation.^1,3,9,10^ Meningeal involvement presents similarly to neoplastic or granulomatous disease and looks similar on brain imaging.^6^ Last, the degenerative lesion occurs due to demyelination and can cause cognitive deterioration and cerebellar or brainstem syndromes, and must be differentiated from multiple sclerosis. Cerebral spinal fluid analysis is usually normal, but may show increased protein and/or cellularity. Imaging findings are nonspecific. In one cohort of patients with ECD, lesions were most commonly located within the hypothalamic-pituitary axis (16 patients, 53%), followed by the facial and skull bones (24 patients, 80%) and meninges (7 patients, 23%).^9^ The distribution of CNS lesions include widespread nodules or masses within the brain parenchyma, tumors or nodular thickening of the dura mater, or a combination of the two. Histologic examination usually shows extensive loss of the myelin sheath with sparing of axons, gliosis, and the development of capillaries along with giant cells loaded with fat and iron granules. In a series of 66 cases of ECD, of whom 22 (33%) had neurological symptoms as the initial presentation, the average time to develop these symptoms was 5 years, including seizures as the presenting symptom in 8 patients (12%).^12^ On the other hand, 4 patients (6%) were asymptomatic but had abnormal MRI findings, which result suggests that brain imaging is important in the assessment of patients with ECD. Orbital infiltration occurs in 25% of cases, causing exophthalmos, which occasionally requires surgical debulking.^1,6^

Because of the rarity of this disease and a lack of randomized controlled trials, there is no evidence-based treatment for ECD. One treatment with possible benefit is IFN-α or PEGylated IFN-α. The clinical response to IFN-α is variable, and dose and duration of therapy are unknown, but in general, disease burden and survival improve with treatment.^2,6^ Corticosteroid treatment reduces inflammation rapidly, but is not effective long-term as monotherapy. Based on small reports, other treatment options include anticytokines, cytotoxic agents, and bisphosphonates. Preliminary studies using vemurafenib, an inhibitor of BRAF harboring the V600E mutation, has shown promising results, mainly in refractory cases.^1,3,6,13^ In cases of localized brain involvement with meningioma-like tumors, surgical resection should be considered. However, such tumors may recur, and almost all patients with neurological symptoms develop progressive disability. Prognosis and clinical course are related to degree of visceral involvement, with CNS involvement one of the strongest independent predictors of mortality. The general survival rate for patients with ECD at 1 and 5 years are 96% and 68%, respectively.^3^ The most common cause of death is lung fibrosis, renal failure secondary to retroperitoneal involvement, and heart failure.

In summary, we describe an atypical case of ECD. In general, the presence of bone pain and diabetes insipidus, neither of which were present at the outset in our case, along with associated inflammatory markers and histology, should raise suspicion for ECD. Most cases reported in the literature thus far debuted as osteosclerosis, either with or without symptoms. Our patient, conversely, presented with late and asymptomatic bone involvement. In most cases of ECD, diagnosis is delayed because of the heterogeneity of clinical manifestations and the need for multiple biopsies.
